# A New Extended Soft Intersection Set to (*M*, *N*)-*SI* Implicative Fitters of *BL*-Algebras

**DOI:** 10.1155/2014/517039

**Published:** 2014-07-21

**Authors:** Jianming Zhan, Qi Liu, Hee Sik Kim

**Affiliations:** ^1^Department of Mathematics, Hubei Minzu University, Enshi, Hubei 445000, China; ^2^Department of Mathematics, Hanyang University, Seoul 133-791, Republic of Korea

## Abstract

Molodtsov's soft set theory provides a general mathematical framework for dealing with uncertainty. The concepts of (*M*, *N*)-*SI* implicative (Boolean) filters of *BL*-algebras are introduced. Some good examples are explored. The relationships between (*M*, *N*)-*SI* filters and (*M*, *N*)-*SI* implicative filters are discussed. Some properties of (*M*, *N*)-*SI* implicative (Boolean) filters are investigated. In particular, we show that (*M*, *N*)-*SI* implicative filters and (*M*, *N*)-*SI* Boolean filters are equivalent.

## 1. Introduction

We know that dealing with uncertainties is a major problem in many areas such as economics, engineering, medical sciences, and information science. These kinds of problems cannot be dealt with by classical methods because some classical methods have inherent difficulties. To overcome them, Molodtsov [1] introduced the concept of a soft set as a new mathematical tool for dealing with uncertainties that is free from the difficulties that have troubled the usual theoretical approaches. Since then, especially soft set operations have undergone tremendous studies; for examples, see [2–5]. At the same time, soft set theory has been applied to algebraic structures, such as [6–8]. We also note that soft set theory emphasizes balanced coverage of both theory and practice. Nowadays, it has promoted a breath of the discipline of information sciences, decision support systems, knowledge systems, decision-making, and so on; see [9–13].

*BL*-algebras, which have been introduced by Hájek [14] as algebraic structures of basic logic, arise naturally in the analysis of the proof theory of propositional fuzzy logic. Turunen [15] proposed the concepts of implicative filters and Boolean filters in *BL*-algebras. Liu et al. [16, 17] applied fuzzy set theory to *BL*-algebras. After that, some researchers have further investigated some properties of *BL*-algebras. Further, Ma et al. investigated some kinds of generalized fuzzy filters *BL*-algebras and obtained some important results; see [18, 19]. Zhang et al. [20, 21] described the relations between pseudo-BL, pseudo-effect algebras, and BCC-algebras, respectively. The other related results can be found in [22, 23].

Recently, Çağman et al. put forward soft intersection theory; see [24, 25]. Jun and Lee [26] applied this theory to *BL*-algebras. Ma and Kim [27] introduced a new concept: (*M*, *N*)-soft intersection set. They introduced the concept of (*M*, *N*)-soft intersection filters of *BL*-algebras and investigated some related properties.

In this paper, we introduce the concept of (*M*, *N*)-soft intersection implicative filters of *BL*-algebras. Some related properties are investigated. In particular, we show that (*M*, *N*)-*SI* implicative filters and (*M*, *N*)-*SI* Boolean filters are equivalent.

## 2. Preliminaries

Recall that an algebra *L* = (*L*, ≤, ∧, ∨, ⊙, →, 0,1) is a *BL*-*algebra* [14] if it is a bounded lattice such that the following conditions are satisfied:

- (*L*, ⊙, 1) is a commutative monoid,
- ⊙ and → form an adjoin pair, that is, *z* ≤ *x* → *y* if and only if *x*⊙*z* ≤ *y* for all *x*, *y*, *z* ∈ *L*,
- *x*∧*y* = *x*⊙(*x* → *y*),
- (*x* → *y*)∨(*y* → *x*) = 1.

In what follows, *L* is a *BL*-algebra unless otherwise is specified.

In any *BL*-algebra *L*, the following statements are true (see [14, 15]):

(*a_1_*) *x* ≤ *y*⇔*x* → *y* = 1,
(*a_2_*) *x* → (*y* → *z*) = (*x*⊙*y*) → *z* = *y* → (*x* → *z*),
(*a_3_*) *x*⊙*y* ≤ *x*∧*y*,
(*a_4_*) *x* → *y* ≤ (*z* → *x*)→(*z* → *y*), *x* → *y* ≤ (*y* → *z*)→(*x* → *z*),
(*a_5_*) *x* → *x*′ = *x*′′ → *x*,
(*a_6_*) *x*∨*x*′ = 1⇒*x*∧*x*′ = 0,
(*a_7_*) (*x* → *y*)⊙(*y* → *z*) ≤ *x* → *z*,
(*a_8_*) *x* ≤ *y*⇒*x* → *z* ≥ *y* → *z*,
(*a_9_*) *x* ≤ *y*⇒*z* → *x* ≤ *z* → *y*,
(*a_10_*) *x*∨*y* = ((*x* → *y*) → *y*)∧((*y* → *x*) → *x*),

where *x*′ = *x* → 0.

A nonempty subset *A* of *L* is called a* filter* of *L* if it satisfies the following conditions: (I1) 1 ∈ *A*, (I2) for all *x* ∈ *A*, for all *y* ∈ *L*, *x* → *y* ∈ *A*⇒*y* ∈ *A*.

It is easy to check that a nonempty subset *A* of *L* is a filter of *L* if and only if it satisfies (I3) for all *x*, *y* ∈ *L*, *x*⊙*y* ∈ *A*, (I4) for all *x* ∈ *A*, for all *y* ∈ *L*, *x* ≤ *y*⇒*y* ∈ *A* (see [15]).

Now, we call a nonempty subset *A* of *L* an implicative filter if it satisfies (I1) and (I5) *x* → (*z*′ → *y*) ∈ *A*, *y* → *z* ∈ *A*⇒*x* → *z* ∈ *A*.

A nonempty subset *A* of *L* is said to be a Boolean filter of *L* if it satisfies *x*∨*x*′ ∈ *A*, for all *x* ∈ *A*. (see [15–18]).

From now on, we let *L* be an *BL*-algebra, *U* an initial universe, *E* a set of parameters, *P*(*U*) the power set of *U*, and *A*, *B*, *C*⊆*E*. We let *∅*⊆*M* ⊂ *N*⊆*U*.

**Definition 1 (see [1]).**

A soft set *f*_*A*_ over *U* is a set defined by *f*_*A*_ : *E* → *P*(*U*) such that *f*_*A*_(*x*) = *∅* if *x* ∉ *A*. Here *f*_*A*_ is also called an approximate function. A soft set over *U* can be represented by the set of ordered pairs *f*_*A*_ = {(*x*, *f*_*A*_(*x*))∣*x* ∈ *E*, *f*_*A*_(*x*) ∈ *P*(*U*)}. It is clear to see that a soft set is a parameterized family of subsets of *U*. Note that the set of all soft sets over *U* will be denoted by *S*(*U*).

**Definition 2 (see [9]).**

Let *f*_*A*_, *f*_*B*_ ∈ *S*(*U*).

(1) *f*_*A*_ is said to be a soft subset of *f*_*B*_ and denoted by $f_{A}\tilde{\subseteq }f_{B}$ if *f*_*A*_(*x*)⊆*f*_*B*_(*x*), for all *x* ∈ *E*. *f*_*A*_ and *f*_*B*_ are said to be soft equally, denoted by *f*_*A*_ = *f*_*B*_, if $f_{A}\tilde{\subseteq }f_{B}$ and $f_{A}\tilde{⊇}f_{B}$.
(2) The union of *f*_*A*_ and *f*_*B*_, denoted by $f_{A}\tilde{\cup }f_{B}$, is defined as $f_{A}\tilde{\cup }f_{B}=f_{A\cup B}$, where *f*_*A*∪*B*_(*x*) = *f*_*A*_(*x*) ∪ *f*_*B*_(*x*), for all *x* ∈ *E*.
(3) The intersection of *f*_*A*_ and *f*_*B*_, denoted by $f_{A}\tilde{\cap }f_{B}$, is defined as $f_{A}\tilde{\cap }f_{B}=f_{A\cap B}$, where *f*_*A*∩*B*_(*x*) = *f*_*A*_(*x*)∩*f*_*B*_(*x*), for all *x* ∈ *E*.

**Definition 3 (see [26]).**

(1) A soft set *f*_*L*_ over *U* is called an *SI*- filter of *L* over *U* if it satisfies

(*S_1_*) *f*_*L*_(*x*)⊆*f*_*L*_(1) for any *x* ∈ *L*,
(*S_2_*) *f*_*L*_(*x* → *y*)∩*f*_*L*_(*x*)⊆*f*_*L*_(*y*) for all *x*, *y* ∈ *L*.

(2) A soft set *f*_*L*_ over *U* is called an *SI*-*implicative filter* of *L* over *U* if it satisfies (*S*_1_) and

(*S_3_*) *f*_*L*_(*x* → (*z*′ → *y*))∩*f*_*l*_(*y* → *z*)⊆*f*_*L*_(*x* → *z*), for all *x*, *y*, *z* ∈ *L*.

In [27], Ma and Kim introduced the concept of (*M*, *N*)-*SI* filters in *BL*-algebras.

**Definition 4 (see [27]).**

A soft set *f*_*S*_ over *U* is called an (*M*, *N*)-soft intersection filter (briefly, (*M*, *N*)-*SI* filter) of *L* over *U* if it satisfies

(*SI_1_*) *f*_*L*_(*x*)∩*N*⊆*f*_*L*_(1) ∪ *M* for all *x* ∈ *L*,
(*SI_2_*) *f*_*L*_(*x* → *y*)∩*f*_*L*_(*x*)∩*N*⊆*f*_*L*_(*y*) ∪ *M* for all *x*, *y* ∈ *L*.

Define an ordered relation “${\tilde{\subseteq }}_{\left(M,N\right)}$” on *S*(*U*) as follows. For any *f*_*L*_, *g*_*L*_ ∈ *S*(*U*), *∅*⊆*M* ⊂ *N*⊆*U*, we define $f_{L}{\tilde{\subseteq }}_{\left(M,N\right)}g_{L}\Leftrightarrow f_{L}\cap N{\tilde{\subseteq }}_{\left(M,N\right)}g_{L}\cup M$.

And we define a relation “=_(*M*,*N*)_” as follows: $f_{L}{=}_{\left(M,N\right)}g_{L}\Leftrightarrow f_{L}{\tilde{\subseteq }}_{\left(M,N\right)}g_{L}$ and $g_{L}{\tilde{\subseteq }}_{\left(M,N\right)}f_{L}$.

**Definition 5 (see [27]).**

A soft set *f*_*S*_ over *U* is called an (*M*, *N*)-soft intersection filter (briefly, (*M*, *N*)-*SI* filter) of *L* over *U* if it satisfies

(*SI_1_′*) $f_{L}(x){\tilde{\subseteq }}_{\left(M,N\right)}f_{L}(1)$ for all *x* ∈ *L*,
(*SI_2_′*) $f_{L}(x\to y)\cap f_{L}(x){\tilde{\subseteq }}_{\left(M,N\right)}f_{L}(y)$ for all *x*, *y* ∈ *L*.

## 3. (*M*, *N*)-*SI* Implicative (Boolean) Filters

In this section, we investigate some characterizations of (*M*, *N*)-*SI* implicative filters of *BL*-algebras. Finally, we prove that a soft set in *BL*-algebras is an (*M*, *N*)-*SI* implicative filter if and only if it is an (*M*, *N*)-*SI* Boolean filter.

**Definition 6 .**

A soft set *f*_*L*_ over *U* is called an (*M*, *N*)-soft intersection implicative filter (briefly, (*M*, *N*)-*SI* implicative filter) of *L* over *U* if it satisfies (*SI*_1_) and (*SI*_3_) $f_{L}(x\to (z^{\prime }\to y))\cap f_{L}(y\to z)\cap N{\tilde{\subseteq }}_{\left(M,N\right)}f_{L}(y\to z)\cup M$ for all *x*, *y*, *z* ∈ *L*.

**Remark 7 .**

If *f*_*L*_ is an (*M*, *N*)-*SI* implicative filter of *L* over *U*, then *f*_*L*_ is an (*∅*, *U*)-*SI* implicative filter of *L*. Hence every *SI*-implicative filter of *L* is an (*M*, *N*)-*SI* implicative filter of *L*, but the converse need not be true in general. See the following example.

**Example 8 .**

Assume that *U* = *D*_2_ = {〈*x*, *y*〉∣*x* ^2^ = *y* ^2^ = *e*, *xy* = *yx*} = {*e*, *x*, *y*, *yx*}, dihedral group, is the universe set.

Let *L* = {0, *a*, *b*, 1}, where 0 < *a* < *b* < 1. Then we define *x*∧*y* = min⁡{*x*, *y*}, *x*∨*y* = max⁡{*x*, *y*} and ⊙ and → as follows:

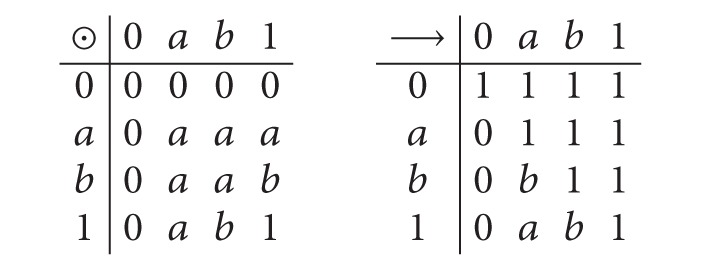
(1)
Then (*L*, ∧, ∨, ⊙, →, 1) is a *BL*-algebra.

Let *M* = {*e*, *y*} and *N* = {*e*, *x*, *y*}.

Define a soft set *f*_*L*_ over *U* by *f*_*L*_(1) = {*e*, *x*}, *f*_*L*_(*a*) = *f*_*L*_(*b*) = {*e*, *x*, *y*}, and *f*_*L*_(0) = {*e*, *y*}. Then one can easily check that *f*_*L*_ is an (*M*, *N*)-*SI* implicative filter of *L* over *U*, but it is not an *SI* implicative filter of *L* over *U* since *f*_*L*_(1) = {*e*, *x*}⊉*f*_*L*_(*a*).

By means of “${\tilde{\subseteq }}_{\left(M,N\right)}$,” we can obtain the following equivalent concept.

**Definition 9 .**

A soft set *f*_*L*_ over *U* is called an (*M*, *N*)-*SI* implicative filter of *L* over *U* if it satisfies (*SI*_1_′) and (*SI*_3_′) $f_{L}(x\to (z^{\prime }\to y))\cap f_{L}(y\to z){\tilde{\subseteq }}_{\left(M,N\right)}f_{L}(y\to z)$ for all *x*, *y*, *z* ∈ *L*.

From the above definitions, we have the following.

**Proposition 10 .**

Every (*M*, *N*)-*SI* implicative filter of *L* over *U* is an (*M*, *N*)-*SI* filter, but the converse may not be true as shown in the following example.

**Example 11 .**

Define *x*⊙*y* = min⁡{*x*, *y*} and

(2) $$\begin{array}{c} x⟶y=\{\begin{array}{cc} 1, & \text{if}\;x\leq y,\\ y, & \text{if}\;x>y. \end{array} \end{array}$$

Then *L* = ([0,1], ∧, ∨, ⊙, →, 0,1) is a *BL*-algebra.

Let *U* = *L*, *M* = {0.5,0.75}, and *N* = {0.5,0.75,1}.

Define a soft set *f*_*L*_ over *U* by

(3) $$\begin{array}{c} f_{L}\left(x\right)=\{\begin{array}{cc} \left\{0,0.5\right\}, & \text{if}\;x\in \left[0,\frac{1}{2}\right],\\ \left\{0.5,1\right\}, & \text{if}\;x\in \left[\frac{1}{2},1\right]. \end{array} \end{array}$$

Then one can easily check that *f*_*L*_ is an (*M*, *N*)-*SI* filter of *L* over *U*, but it is not an (*M*, *N*)-*SI* implicative filter of *L* over *U*. Since *f*_*L*_(2/3 → ((1/3)′ → 1/4))∩*f*_*L*_(1/4 → 1/3)∩*N* = *f*_*L*_(1)∩*f*_*L*_(1)∩*N* = {0.5,1}∩{0.5,0.75,1} = {0.5,1} and *f*_*L*_(2/3 → 1/4) ∪ *M* = *f*_*L*_(1/4) ∪ *M* = {0,0.5}∪{0.5,0.75} = {0,0.5,0.75}, this implies that *f*_*L*_(2/3 → ((1/3)′ → 1/4))∩*f*_*L*_(1/4 → 1/3)∩*N*⊈*f*_*L*_(*x* → *z*) ∪ *M*.

**Lemma 12 (see [27]).**

If a soft set *f*_*L*_ over *U* is an (*M*, *N*)-*SI* filter of *L*, then for any *x*, *y*, *z* ∈ *L* we have

1. $x\leq y\Rightarrow f_{L}(x){\tilde{\subseteq }}_{\left(M,N\right)}f_{L}(y)$,
2. $f_{L}(x\to y)=f_{L}(1)\Rightarrow f_{L}(x){\tilde{⊈}}_{\left(M,N\right)}f_{L}(y)$,
3. *f*_*L*_(*x*⊙*y*)=_(*M*,*N*)_ *f*_*L*_(*x*)∩*f*_*L*_(*y*)=_(*M*,*N*)_ *f*_*L*_(*x*∧*y*),
4. *f*_*L*_(0)=_(*M*,*N*)_ *f*_*L*_(*x*)∩*f*_*L*_(*x*′),
5. $f_{L}(x\to y)\cap f_{L}(y\to z){\tilde{\subseteq }}_{\left(M,N\right)}f_{L}(x\to z)$,
6. $f_{L}(x)\cap f_{L}(y){\tilde{\subseteq }}_{\left(M,N\right)}f_{L}(y⊙z\to y⊙z)$,
7. $f_{L}(x\to y){\tilde{\subseteq }}_{\left(M,N\right)}f_{L}((y\to z)\to (x\to z))$,
8. $f_{L}(x\to y){\tilde{\subseteq }}_{\left(M,N\right)}f_{L}((z\to x)\to (z\to y))$.

**Theorem 13 .**

Let *f*_*L*_ be an (*M*, *N*)-*SI* filter of *L* over *U*, then the following are equivalent:

1. *f*_*L*_ is an (*M*, *N*)-*SI* implicative filter of *L*,
2. $f_{L}(x\to z){\tilde{⊇}}_{\left(M,N\right)}f_{L}(x\to (z^{\prime }\to z))$, for all *x*, *y*, *z* ∈ *L*,
3. *f*_*L*_(*x* → *z*)=_(*M*,*N*)_ *f*_*L*_(*x* → (*z*′ → *z*)), for all *x*, *y*, *z* ∈ *L*,
4. $f_{L}(x\to z){\tilde{⊇}}_{\left(M,N\right)}f_{L}(y\to (x\to (z^{\prime }\to z)))\cap f_{L}(y)$, for all *x*, *y*, *z* ∈ *L*.

**Proof**

(1) ⇒ (2) Assume that *f*_*L*_ is an (*M*, *N*)-*SI* filter of *L* over *U*. Putting *y* = *z* in (*SI*_3_), then

(4) fL(x⟶z)∪M =(fL(x⟶z)∪M)∩M ⊇(fL(x⟶(z′⟶z))∩fL(z⟶z)∩N)∪M =(fL(x⟶(z′⟶z))∩fL(1)∩N)∪M ⊇fL(x⟶(z′⟶z))∩(fL(1)∪M)∩N ⊇fL(x⟶(z′⟶z))∩N;

that is, $f_{L}(x\to z){\tilde{⊇}}_{\left(M,N\right)}f_{L}(x\to (z^{\prime }\to z))$. Thus, (2) holds.

(2) ⇒ (3) By (*a*_1_) and (*a*_2_), *x* → *z* ≤ *z*′ → (*x* → *z*) = *x* → (*z*′ → *z*); then it follows from Lemma 12 (1) that $f_{L}(x\to z){\tilde{\subseteq }}_{\left(M,N\right)}f_{L}(x\to (z^{\prime }\to z))$. Thus, (3) holds.

(3) ⇒ (4) Assume that (4) holds. By Lemma 12 (5), we have $f_{L}(x⊙z^{\prime }\to y)\cap f_{L}(y\to z){\tilde{\subseteq }}_{\left(M,N\right)}f_{L}(x⊙z^{\prime }\to z)$. By (*a*_2_), $f_{L}(x\to (z^{\prime }\to y))\cap f_{L}(y\to z){\tilde{\subseteq }}_{\left(M,N\right)}f_{L}(x\to (z^{\prime }\to z))$.

(4) ⇒(1) Putting *y* = 1 in (4), we have

(5) $$\begin{array}{c} f_{L}\left(x⟶z\right){\tilde{⊇}}_{\left(M,N\right)}f_{L}\left(x⟶\left(z^{\prime }⟶z\right)\right). \end{array}$$

Hence

(6) $$\begin{array}{c} f_{L}\left(z⟶z\right){\tilde{⊇}}_{\left(M,N\right)}f_{L}\left(x⟶\left(z^{\prime }⟶y\right)\right)\cap f_{L}\left(y⟶z\right). \end{array}$$

Thus, (*SI*_3_) holds. This shows that *f*_*L*_ is an (*M*, *N*)-*SI* implicative filter of *L* over *U*.

Now, we introduce the concept of (*M*, *N*)-*SI* Boolean filters of *BL*-algebras.

**Definition 14 .**

Let *f*_*L*_ be an (*M*, *N*)-*SI* filter of *L* over *U*, then *f*_*L*_ is called an (*M*, *N*)-*SI* Boolean filter of *L* over *U* if it satisfies

(*SI_4_*) *f*_*L*_(*x*∨*x*′)=_(*M*,*N*)_ *f*_*L*_(1) for all *x* ∈ *L*.

**Theorem 15 .**

A soft set *f*_*L*_ over *U* is an (*M*, *N*)-*SI* implicative filter of *L* if and only if it is an (*M*, *N*)-*SI* Boolean filter.

**Proof**

Assume that *f*_*L*_ over *U* is an (*M*, *N*)-*SI* Boolean filter of *L* over *U*. Then

(7) fL(x⟶z) ⊇~(M,N)fL((z∨z′)⟶(x⟶z))∩fL(z∨z′) =(M,N)fL((z∨z′)⟶(x⟶z))∩fL(1) ⊇~(M,N)fL((z∨z′)⟶(x⟶z)).

By (*a*_10_) and (*a*_1_), we have

(8) (z∨z′)⟶(x→z) =(z⟶(x⟶z))∧(z′⟶(x⟶z)) =z′⟶(x⟶z)=x⟶(z′⟶z).

Hence $f_{L}(x\to z){\tilde{⊇}}_{\left(M,N\right)}f_{L}(x\to (z^{\prime }\to z))$. It follows from Theorem 13 that *f*_*L*_ is an (*M*, *N*)-*SI* implicative filter of *L* over *U*.

Conversely, assume that *f*_*L*_ is an (*M*, *N*)-*SI* implicative filter of *L* over *U*. By Theorem 13, we have

(9) fL((x′⟶x)⟶x) =(M,N)fL((x′⟶x)⟶(x′→x))=fL(1),fL((x⟶x′)⟶x′) =(M,N)fL((x⟶x′)⟶(x′′⟶x′)) =fL((x⟶x′)⟶(x⟶x))=fL(1).

By Lemma 12, we have

(10) fL(x∨x′) =(M,N)fL((x⟶x′)⟶x′)∩fL((x′⟶x)⟶x) =(M,N)fL(1).

Hence *f*_*L*_ is an (*M*, *N*)-*SI* Boolean filter of *L* over *U*.

**Remark 16 .**

Every (*M*, *N*)-*SI* implicative filter and (*M*, *N*)-*SI* Boolean filter in *BL*-algebras are equivalent.

Next, we give some characterizations of (*M*, *N*)-*SI* implicative (Boolean) filters in *BL*-algebras.

**Theorem 17 .**

Let *f*_*L*_ be an (*M*, *N*)-*SI* filter of *L* over *U*, then the following are equivalent:

1. *f*_*L*_ is an (*M*, *N*)-*SI* implicative (Boolean) filter,
2. *f*_*L*_(*x*)=_(*M*,*N*)_ *f*_*L*_(*x*′ → *x*), for all *x* ∈ *L*,
3. *f*_*L*_((*x* → *y*) → *x*)⊆_(*M*,*N*)_ *f*_*L*_(*x*), for all *x*, *y* ∈ *L*,
4. *f*_*L*_((*x* → *y*) → *x*)=_(*M*,*N*)_ *f*_*L*_(*x*), for all *x*, *y* ∈ *L*,
5. $f_{L}(x){\tilde{⊇}}_{\left(M,N\right)}f_{L}(z\to ((x\to y)\to x))\cap f_{L}(z)$, for all *x*, *y*, *z* ∈ *L*.

**Proof**

(1) ⇒ (2). Assume that *f*_*L*_ is an (*M*, *N*)-*SI* implicative (Boolean) filter of *L* over *U*. By Theorem 13, we have

(11) fL(x)=fL(1⟶x)=(M,N)fL(1→(x′⟶x))=fL(x′⟶x).

Thus, (2) holds.

(2) ⇒ (3). By (*a*_1_), (*a*_2_), and (*a*_8_), we have *x*′ ≤ *x* → *y* and so (*x* → *y*) → *x* ≤ *x*′ → *x*. By Lemma 12, $f_{L}((x\to y)\to x){\tilde{\subseteq }}_{\left(M,N\right)}f_{L}(x^{\prime }\to x)$. Combining (2), $f_{L}(x){=}_{\left(M,N\right)}f_{L}(x^{\prime }\to x){\tilde{⊇}}_{\left(M,N\right)}f_{L}((x\to y)\to x)$. Thus, (3) holds.

(3) ⇒ (4). Since *x* ≤ (*x* → *y*) → *x*, then by Lemma 12 $f_{L}(x){\tilde{\subseteq }}_{\left(M,N\right)}f_{L}((x\to y)\to x)$. Combining (3), *f*_*L*_(*x*)=_(*M*,*N*)_ *f*_*L*_((*x* → *y*) → *x*).

(4) ⇒ (5). By (*SI*_2_), $f_{L}((x\to y)\to x){\tilde{⊇}}_{\left(M,N\right)}f_{L}(z\to ((x\to y)\to x))\cap f_{L}(z)$. Combining (4), we have $f_{L}(x){\tilde{⊇}}_{\left(M,N\right)}f_{L}(z\to ((x\to y)\to x))\cap f_{L}(z)$. Thus, (5) holds.

(5) ⇒ (1). By (*a*_1_), *z* ≤ *x* → *z*. By (*a*_8_), (*x*→*z*)′ ≤ *z*′ and so *z*′ → (*x* → *z*) ≤ (*x*→*z*)′ → (*x* → *z*). Then by Lemma 12, $f_{L}(z^{\prime }\to (x\to z)){\tilde{\subseteq }}_{\left(M,N\right)}f_{L}({\left(x\to z\right)}^{\prime }\to (x\to z)){=}_{\left(M,N\right)}f_{L}(1\to ({\left(x\to z\right)}^{\prime }\to (x\to z)))\cap f_{L}(1)$. By (5), $f_{L}(x\to z){\tilde{⊇}}_{\left(M,N\right)}f_{L}(z^{\prime }\to (x\to z))$ and so $f_{L}(x\to z){\tilde{⊇}}_{\left(M,N\right)}f_{L}(x\to (z^{\prime }\to z))$. Therefore, it follows from Theorem 13 that *f*_*L*_ is an (*M*, *N*)-*SI* implicative filter of *L*.

Finally, we investigate extension properties of (*M*, *N*)-*SI* implicative filters of *BL*-algebras.

**Theorem 18 (extension property).**

Let *f*_*L*_ and *g*_*L*_ be two (*M*, *N*)-*SI* filters of *L* over *U* such that *f*_*L*_(1)=_(*M*,*N*)_ *g*_*L*_(1) and $f_{L}(x){\tilde{\subseteq }}_{\left(M,N\right)}g_{L}(x)$ for all *x* ∈ *L*. If *f*_*L*_ is an (*M*, *N*)-*SI* implicative (Boolean) filter of *L*, then so is *g*_*L*_.

**Proof**

Assuming that *f*_*L*_ is an (*M*, *N*)-*SI* implicative (Boolean) filter of *L* over *U*, then *f*_*L*_(*x*∨*x*′)=_(*M*,*N*)_ *f*_*L*_(1) for all *x* ∈ *L*. By hypothesis, $g_{L}(x\vee x^{\prime }){\tilde{⊇}}_{\left(M,N\right)}f_{L}(x\vee x^{\prime }){=}_{\left(M,N\right)}f_{L}(1){=}_{\left(M,N\right)}g_{L}(1)$. By (*SI*_1_′), we have $g_{L}(1){\tilde{⊇}}_{\left(M,N\right)}g_{L}(x\vee x^{\prime })$. Thus, *g*_*L*_(*x*∨*x*′)=_(*M*,*N*)_ *g*_*L*_(1). Hence *g*_*L*_ is an (*M*, *N*)-*SI* implicative (Boolean) filter of *L*.

## 4. Conclusions

In this paper, we introduce the concepts of (*M*, *N*)-*SI* implicative filters and (*M*, *N*)-*SI* Boolean filters of *BL*-algebras. Then we show that every (*M*, *N*)-*SI* Boolean filter is equivalent to (*M*, *N*)-*SI* implicative filters. In particular, some equivalent conditions for (*M*, *N*)-*SI* Boolean filters are obtained. We hope it can lay a foundation for providing a new soft algebraic tool in many uncertainties problems.

To extend this work, one can apply this theory to other fields, such as algebras, topology, and other mathematical branches. To promote this work, we can further investigate (*M*, *N*)-*SI* prime (semiprime) Boolean filters of *BL*-algebras. Maybe one can apply this idea to decision-making, data analysis, and knowledge based systems.
